# Comminution of Dry Lignocellulosic Biomass: Part II. Technologies, Improvement of Milling Performances, and Security Issues

**DOI:** 10.3390/bioengineering5030050

**Published:** 2018-06-22

**Authors:** Claire Mayer-Laigle, Rova Karine Rajaonarivony, Nicolas Blanc, Xavier Rouau

**Affiliations:** UMR Ingénierie des Agropolymères et des Technologies Emergentes (IATE), University of Montpellier, CIRAD, INRA, Montpellier SupAgro, 34060 Montpellier CEDEX 01, France; karine.rajaonarivony@supagro.fr (R.K.R.); nicolas.blanc@inra.fr (N.B.); xavier.rouau@inra.fr (X.R.)

**Keywords:** milling, plant materials, grinding, energy consumption, torrefaction, cryogenic milling, atex explosion hazard

## Abstract

Lignocellulosic feedstocks present a growing interest in many industrial processes as they are an ecological alternative to petroleum-based products. Generally, the size of plant raw materials needs to be reduced by milling step(s), to increase density, facilitate transport and storage, and to increase reactivity. However, this unit operation can prove to be important in term of investments, functioning costs, and energy consumption if the process is not fully adapted to the histological structure of the plant material, possibly challenging the profitability of the whole chain of the biomass conversion. In this paper, the different technologies that can be used for the milling of lignocellulosic biomass were reviewed and different avenues are suggested to improve the milling performances thanks to thermal pretreatments. Based on examples on wheat straw milling, the main points to take into consideration in the choice of a milling technologies have been highlighted in regards to the specifications of ground powder. A specific focus on the hazards associated to the milling and the manipulation of fine biomass particles is also realized at the end of the paper from the perspective of industrial applications.

## 1. Introduction

Since the dawn of the time, wood has been used as a source of energy (for heating) and building matderials. From the industrial revolution until the end of the nineteenth century, its use has progressively been replaced by coal, then petroleum, for energetic application and technical materials. The decrease of the fossil resources leads to a reconsideration of all types of biomass as a renewable source of raw materials. Indeed, the diversity of biomasses and their chemical composition make them particularly suitable for many applications, such as energy, materials, or green chemistry. In most of them, the biomass is first milled to densify it, to reduce the variability, and to adapt its size to the downstream steps of the transformation process. However, the milling step is often a key point in the whole valorization chain as it can consume a high amount of energy and influences the process yield if the properties of the ground particles are not fully controlled.

During grinding, different mechanical stresses are generated by the grinder and they impact the propagation of fracture paths through the lignocellulosic biomass, leading to particles which differ in term of particle size, shape, surface roughness, and rheological properties, as discussed in the part I of this review [1,2,3]. The interaction between the grinder and the lignocellulosic biomass also affects the energy consumption to reach a given particle size [4,5]. However, the energy consumption is also strongly correlated to the device type, its dimensioning, its operating mode, etc. In some cases, a thermic pre-treatment step prior to the grinding step can enable one to reduce the total energy consumption of the comminution and increase the added value of biomass end-products [6,7].

If biomass coarse grinding and associated technologies have been largely studied in particular for wood and timber products (pellets, condensed wood, chipboard panels), fine and ultrafine milling (below 100 micrometers) are still a scarcely explored field from technological and economical points of view [8,9]. At the industrial scale, this is commonly achieved by wet processing leading to the production of effluents that must be treated, and a supplementary step of biomass drying depending on the application [10]. Several studies have explored the ultra-fine milling of biomass at a laboratory-scale [11,12,13] but few manufacturers offer devices specifically designed for the grinding of biomass at a larger scale and the scale-up of this operation remains a technical lock for industrial applications. In addition, there are several hazards related to the milling and handling of vegetal powders which slow down the efforts deployed in this direction.

The aim of this paper is to describe the current state of the art based on literature review and experts’ opinions on the technological devices that can be used for the fine comminution of biomass and their specificities in terms of mechanical stress and energy consumption. Some thermic pretreatments, as a way to reduce the total energy consumption and enhance the properties of the ground powders, are also discussed. Finally, a specific focus about the hazards associated with the milling and the manipulation of fine biomass particles are reported.

## 2. Milling Technologies and Operating Conditions

The milling of biomass involves generally several steps especially when very fine particles are targeted, as the different technologies are not fully appropriate for all scales. For lignocellulosic materials from meter to centimeter dimensions, one speaks about cutting or crushing. For a scale between 1–10 mm, intermediate milling or comminution is generally used, then fine milling for particles between 50–500 µm and ultra-fine milling, below 20 µm. However, the boundaries between the different scales can vary according to the application and are highly dependent on the appreciation of the authors.

### 2.1. Equipement for Fine and Ultrafine Milling of Lignocellulosic Biomass

**Figure 1:**
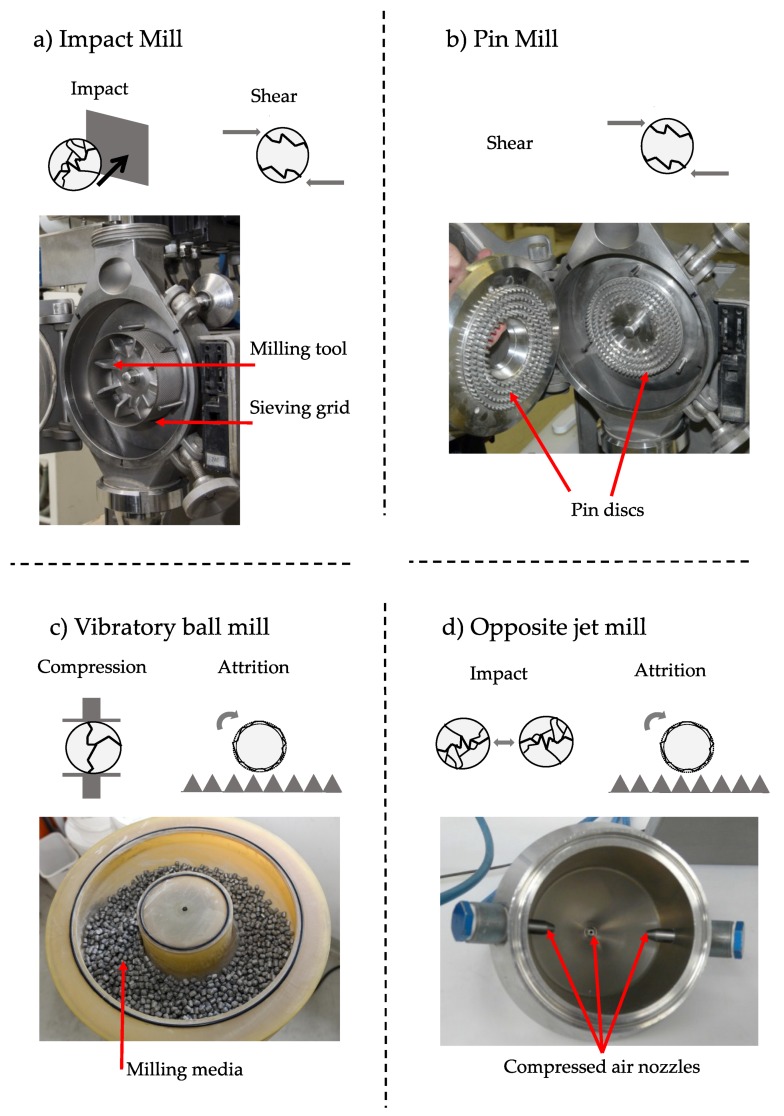
Milling chambers of different devices and their main mechanical stresses: (**a**) impact mill, (**b**) pin mill, (**c**) vibratory ball mill and ppposite jet mill used for fine milling (**a**,**b**), and ultrafine milling (**c**,**d**) of biomass.

The diversity of grinders is as great as the number of manufacturers. Each of them gives a specific name to the grinder and it is better to compare them on the basis of their principles related to the mechanical stress generated inside the device. Figure 1 gives the example of 4 milling chambers of different devices with the aim of highlighting their operating principles. Grinder (a), (b), and (d) are of continuous supply type. The first one (Figure 1a), known as an impact-mill, is constituted of a tool which turns at high speed (between 10,000 and 20,000 rpm) and projects particles against a screen (between 0.1 to 1 mm). This type of grinder has been used for many lignocellulosic materials, such as wheat bran [14], rice straw [15], and wheat straw [16], to obtain particles with a median size between 100 and 500 µm according to the hole sizes of the sieving grids.

In a pin-mill (Figure 1b), the main mechanical stress is shear and is created by a differential speed between the two pin discs. The rotation of the pin discs creates a dynamic selection of the ground particles. When particles are finer than the gap between the pins, impact overrides shear. Several interesting studies show that this type of grinder can be suitable for the fine grinding of wheat bran [17], oat bran [18], and switchgrass [19].

Figure 1c,d shows two grinders that allow one to obtain very fine particles. In the vibratory ball-mill, compression and attrition are created by the milling media (ball and cylpebs) which are maintained in motion by the vibration of the tank [15]. It is more or less equivalent of traditional ball-mill but it is particularly adapted to lignocellulosic biomass [20] by maximizing attrition. In a jet-mill, the particles are projected by air-compressed nozzles in a central point where they collide, leading to their comminution. The size of the ground particles is selected by a rotary sieve operating between 3000 and 20,000 rpm. This grinder allows one to obtain particles below 10 µm but most of the time the particle needs to be pre-ground at an intermediate scale (below 500 µm) [16]. Table 1 summarizes the different operating conditions of the milling equipment shown in Figure 1.

### 2.2. Operating Mode

Grinders can operate in batch or continuous configuration and some of them are commercialized only in one configuration. For others, the operating mode is related to their scales. At lab-scale, most of them operate in batch. At pilot-scale or industrial scale, they operate in continuous supply. In batch mode, it is possible to work with small amounts of raw materials and to efficiently control the cleaning of the grinder when changing the raw materials for various experiments. However, the particle size distributions, even if the mechanical stress is the same, may present significant differences in comparison to the continuous mode. Indeed, in the case of batch mode, the finest particles remain during all the operating time in the grinder chamber, slowing down the comminution kinetics. In addition, for the same median size (d50), the particle size obtained in continuous mode is generally less widely distributed, due to the continuous extraction of the fine particles as soon as they are produced.

## 3. Energy Requirements of the Milling Technologies

The energy consumption increases significantly as the particle size decreases [21]. From a perspective of sustainable technologies, it is important to adjust the target particle size to the intended application. For some applications with high added value, such as building blocks for polymers and materials or molecule extraction for green chemistry, a higher energy expenditure for milling can be afforded, whereas for biomass conversion to energy, it must be kept as low as possible to remain cost-effective.

### 3.1. Examples of Energetic Grinding Laws in Milling Equipment

The particle mechanical strength is, a priori, directly correlated to the milling energy as it has been shown for different type of seeds [22,23]. In the mills where the particles are broken down almost individually, these data can be very useful to model the milling of a material by using population balance models [24]. But, in other mills where interactions between particles are not negligible like in high pressure grinding rolls, these data are insufficient. Some correlation can be observed between the mechanical properties of the lignocellulosic biomass and its behavior during the comminution (energy consumed, kinetics of comminution…) but it is difficult to establish a direct relation [6].

Several grinding laws have been proposed to predict the energy cost of a milling process. The best known are the ones by Von Rittinger, Kick, and Bond. Initially developed for the mineral industry, each of these methods expresses the energy as a function of the variation of the fineness of the product. Von Rittinger [25] states that the specific energy *E_m_* (energy by unit of mass) is directly proportional to the creation of the specific surface area (see Equation (1)) with *K_r_* a constant depending both on the material to grind and the mill, and *S_p_*_1_ and *S_p_*_2_ the specific surfaces of the particle before and after breakage. For lignocellulosic materials, even if it concerns millimeter ranges, this law fits well with data from wood chip, alpha chops, and pellet milling [26,27]. Kick [28] relates later the energy as a function of the ratio of the mean diameter before (*D*_1_) and after milling (*D*_2_) (see Equation (2)). Bond [29] expresses the specific energy as a function of the inverse of the square root of the 80%-by-weight-passing size of the feed and the product (see Equation (3)) with *W_i_* a constant of the material measured in a specific grinding test developed by himself [30]. In general, the Kick’s law is used for coarse grinding, Bond’s for intermediate grinding, and Rittinger’s for fine grinding [31]. From these laws, Bond’s law is the most well-known and the most used in mineral industry for its simplicity.

(1) $$E_{m}=K_{r}\left(S_{p2}-S_{p1}\right)$$

(2) $$E_{m}=K_{k}\ln\left(\frac{D_{1}}{D_{2}}\right)$$

(3) $$E_{m}=10W_{i}\left(\frac{1}{\sqrt{D_{2}}}-\frac{1}{\sqrt{D_{1}}}\right)$$

**Figure 2:**
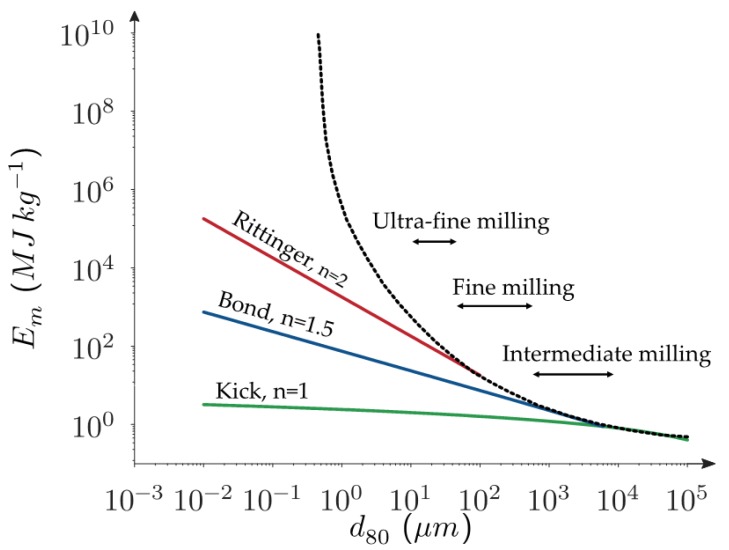
The energy consumption for the intermediate the fine and the ultrafine milling of lignocellulosic biomass and the different grinding laws (Rittinger, Bond, and Kick).

Nevertheless, the discrepancies sometimes appearing between these laws and the reality drove some authors to make some adjustments. Holmes proposed to replace the square root of diameter in Bond’s law by an exponent *r* varying with the material to be milled [32] and Svensson and Murkes discussed the choice of the 80%-by-weight-passing size in Bond’s law [31]. Walker and Shaw suggested to gather Bond, Kick, and Rittinger laws in one based on the fact that all these laws come from one differential equation:

(4) $$dE_{m}=-K_{c}\frac{dD}{D^{n}}$$

with *n* = 1 for Kick’s law, *n* = 1, 5 for Bond’s law, and *n* = 2 for Rittinger’s law. Hukki postulated later that *n* is not constant and depends on the particle size [33], as illustrated in the Figure 2. The aim of this figure is to show that the total energy consumption increases greatly as the particle size decreases and that the different models can fit more or less the energy consumption according to the range of milling considered.

Nevertheless, predictions of the milling energy cost of a given product are difficult to make and a lot of empirical constants are necessary to establish relations between different apparatus and scales [34]. Scale-up procedures are often based on the assertion that the same specific energy consumption leads to the same characteristics of the final product. The comminution of a product resulting from a specific energy is usually measured in a laboratory-scale mill. Mio et al. (2004) have shown that the breakage rate used in population balance models varies linearly with the specific energy input [35]. Thus, the knowledge of this linear law for a material allows one to predict the energy consumption for different scales [36] showing geometric similarities. Indeed, in order to estimate the energy developed by the up-scaled mill, some geometric considerations, such as keeping the same ratio between the length and the diameter of the chamber, must be verified in order to be in the same stress conditions [37]. The mechanical power needed can be determined from a scaling ratio based on these geometric considerations and the increase of feed rate. However, despite taking into account all these elements, the actual energy consumed by the mill may differ from calculations of up-scaling due to variations in the stress distribution or in the heating losses [37]. When it is not possible to have a clear understanding of the behavior change while up-scaling, the procedures rely on empirical data from experimental work [38,39]. As for example, just for ball-mill, up to 8 constants are required to keep *W_i_* constant in Bond’s law [31] depending on the operating conditions and scale of the mill.

### 3.2. Classification of Milling Technologies on the Basis of Energy Requirement

To minimize the total energy consumption, it is important to adapt the mechanical stress generated by the grinder to the structure of the raw material. For very fibrous or filamentous materials, such as flax or hemp, the scutching process allows the separation and the dissociation of fibers from the shives. Similarly, milling involving shear as the main mechanical solicitation is often preferable as pre-milling step as it allows a dissociation of the material by cutting the structure while respecting the plant organization. As a result, the associated energy consumption is less important than for impact processes [40].

However, if the total energy consumption is often a key element in a process selection, it is also important to pay attention to the electrical power of the grinder in regard to the production time of the powder. As an example, milling of biomass around 150–200 µm can be achieved in a ball-mill operating in batch. In this case, the milling time can be important leading to a long-time production and a high total energy consumption in comparison to an impact mill operating in continuous mode. However, ball-mills need low instantaneous electrical power to work, which can be an interesting economical option in the case of stand-alone installation from renewable energy (as solar panel or wind turbine) and if an intermediate storage area is possible.

**Figure 3:**
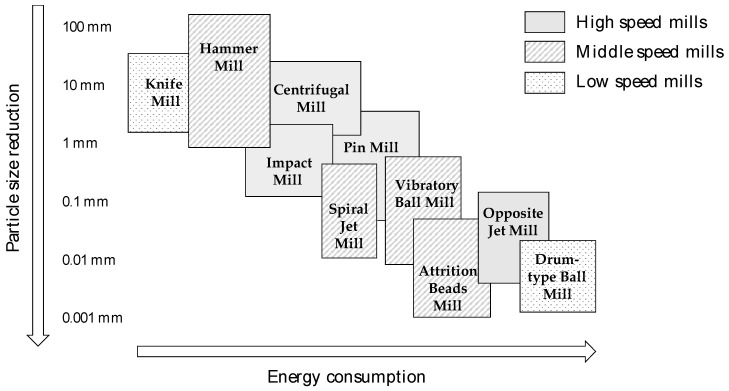
Classification of different milling equipment based on the energy consumption, the particle size reduction capacity, and the speed of milling, from different research works [8,16,21,41,42,43,44,45,46].

As an illustration, Figure 3 shows different grinding technologies ranked according to the milling energy, the particle size that they allow to reach, and the speed of the milling related to the electrical power. Grinders have been ranked in three categories: (i) high speed mills which operate in continuous mode and allow almost instantaneous comminution; (ii) middle speed mills, which are batch mills but with relatively short grinding time (a few minutes to 1 or 2 h according to the targeted size); and (iii) the low speed mills, which are low electrical power mills operating in batch mode but imply longer grinding time (several hours).

This classification is indicative and was realized both on the basis of expert opinions and the compilation of different research works [8,16,21,41,42,43,44,45,46]. However, as previously discussed, it is very difficult to compare the different milling technologies in similar conditions, the exact energetic consumption being dependent on the biomass feedstock properties, the operating conditions, the dimensioning of the facilities, etc. As the different studies used for this compilation were realized with equipment of different sizing and with different biomasses, the aim of Figure 3 is more to give an idea of the range of the energy consumption according to the technology used and the particle size reduction that they allow than to give quantitative data on energy consumption for each grinder with a given biomass.

Two points may be noticed: (i) whatever the milling equipment used, the total energy consumption increases as the particle size decreases; (ii) several milling devices allow one to obtain the targeted particle size with similar energy consumption but with different speeds, related to their electrical power. This is the case, for example, for opposite jet mills and drum-type ball mills. In the first one, it is possible to obtain a particle size less than 10 µm in a very short time, whereas in the drum-type ball mill, the grinding can take more than 200 h [16].

## 4. Thermic Pretreatment Prior to Milling

A pretreatment step either prior to or during the grinding can also be added in the processing chain in order to weaken the plant materials and reduce the total energy consumption. There are several possible methods of pretreatment to modify the material in order to make it more suitable to size reduction with lower energy input. Biological (microorganisms), biochemical (enzymes), chemical (acid, alkali, oxidizing reagents), and physical (pressure, temperature, beams…) pretreatments and their combinations are generally used in solid, semi-solid, or liquid conditions to help fast and extensive degradation of lignocellulosic substrates, with concomitant decrease in mechanical resistance to grinding and milling. In this review, the focus is made on thermic pretreatments which are compatible with applications keeping strictly dry conditions. The heat and cold pretreatments will be considered successively.

### 4.1. Heat Treatments of Lignocellulosic Biomass

Heat pre-treatment consists of the submission of the plant raw material to an elevation of temperature for a shorter or longer time, in order to modify its chemical and mechanical properties. The elevation of the temperature is generally achieved thanks to a hot gas flow (air, inert gas…) but some other treatments with, e.g., microwave, have also been reported, especially for wood [47,48].

The heat treatments induce a weight loss linked to the departure of water in a first stage, then, as temperature rises, a vaporization of extractives and a devolatilization of volatiles accompanying the progressive degradation of components like hemicelluloses, cellulose, and lignin for higher temperature. There are different degrees in the severity of heat treatments. For temperatures below 200 °C, there is a simple drying effect with departure of the moisture content of the material. For stronger heat conditions, not only the water is removed but the matter itself undergoes profound chemical transformations. Torrefaction is a thermal treatment at temperatures between 200 and 300 °C under atmospheric pressure in an inert atmosphere. In these conditions, the biomass material is converted into a major solid product together with lower proportions of condensable volatiles and permanent gases. For same treatment conditions, the yield in solid depends on the biomass source, and is related to its hemicellulose content and nature. Indeed, hemicellulose polysaccharides are more susceptible to thermal degradation than cellulose and lignin, and therefore are first affected by the process [49]. The material undergoes a modification of its structure due to some rearrangement of the components like new cross-links in the lignin network [50]. A major change is the relative increase in crystallinity of cellulose caused by the degradation of the hemicelluloses [51]. This phenomenon leads to a more rigid material and influences some parameters like the elasticity and water absorption capacity [52]. As a result of torrefaction process, there are significant differences also in condensable volatiles and gaseous products yields and compositions depending on the nature of the biomass [53].

### 4.2. Effects of Heat Treatments on Mechanical Properties, Grindability, and Grinding Energy

The temperature-induced changes in composition and structure modify certain mechanical properties of the materials quantified by some authors in quasi-static conditions by the Young’s Modulus and the module of rupture [50,54]. Furthermore, it is possible to quantify the effect of a thermal pretreatment by following the energetic consumption of the machine during grinding [55] or the particle size of resulting powders [56]. Extensive drying of the biomass at moderate temperature (<200 °C) has consequences on the mechanical properties by removing the plasticizing effect of water. For example, Tavakoli et al. (2009) showed in playing with relative humidity of wheat straw, an increase in the shear resistance and the shear specific energy, whereas Young’s Modulus decreased, with higher water contents [54]. This is a general trend in the relations between water content and plant-based material mechanical properties. The torrefaction goes further in rendering the material more rigid, brittle, and more sensitive to grinding [57] and the pretreatment also tends to lower the grinding energy consumption [58,59]. Several authors have shown that torrefaction of wood improves the grindability of the material [57,60] and have consequences on the morphology and the size distribution of the particles. This aptitude is linked to the structural modifications inside the material.

The grindability of torrefied woods is therefore improved even by moderate heat conditions (<260 °C) when the plasticizing effect of internal water content is fully removed by drying in depth, but it can be further strongly improved with treatments beyond 260 °C causing irreversible ruptures and degradation in biopolymers [55]. In comparing the grindability of several biomasses torrefied to a same solid weight loss (17%), it was found grinding energy reductions by a factor 2 or 3 for wood species and miscanthus, whereas wheat straw grindability was less improved [53]. However, it is important to keep in mind that heat treatments could also be high energy consuming and it is important to do a comparison of the energy consumption at the scale of the whole process and not only on the milling step.

The energy efficiency of the torrefaction process is the ratio between the energy yield in the product and the total energy contained in the feedstock plus the process energy input [61]. It is estimated that a 90% efficiency can be attained in the torrefaction process, but depending on moisture content of biomass feedstock, 80% efficiency seems more reasonable in practice. The total energy balance may become less favorable with increasing temperatures beyond 260 °C. A balance between energy consumed by torrefaction and decrease in grinding energy must be found. Depending on wood species, an optimum could be established around 10% of weight loss [55]. The efficiency of the process can be appreciably improved in using gaseous and liquid products generated during torrefaction as a heating source [62]. But, sufficient amounts of these products suitable to sustain the reaction can be obtained only in the high range of torrefaction conditions (>260 °C). An alternative to conventionally heated reactors can be the microwave heating of biomass [63]. The advantage is that the bulk material is selectively heated wholeheartedly with rapid water and volatiles release, causing cracks which in turn accelerate heat and mass transfer, which makes this technology energetically attractive. At the industrial scale, however, a major disadvantage is that electricity is required for the microwave generator, which is difficult to produce with acceptable efficiency from the torrefaction gas.

Powders obtained from grinding/milling of torrefied biomass solids will exhibit modified properties, as compared to products from non-torrefied starting materials. These are: dimensional stability, enhanced hydrophobicity, microbial stability, energetic densification, and improved flow properties [7,53].

### 4.3. The Specific Case of Cryogenic Milling

**Figure 4:**
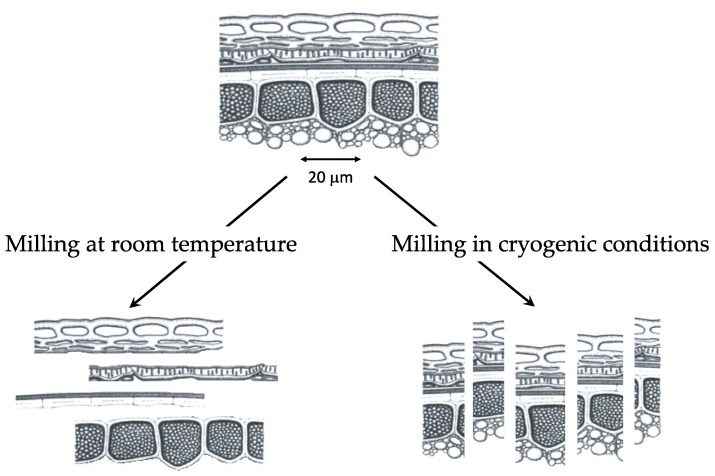
Influence of ambient and cryogenic milling on the dissociation of tissues in the plant materials.

Grinding/milling samples in cryogenic conditions, generally using liquid nitrogen (0 to −196 °C), is a current technique to obtain very finely divided and stabilized vegetal samples for analytical characterization. Low temperature conditions embrittle the materials and also inactivate enzyme activities in fresh plants. At larger scale, a few examples of studies on cold grinding of plant-sourced materials are reported. Hemery et al. (2011) ground wheat bran in a high-speed impact mill operated with a liquid nitrogen-cooled feeder [14]. A 50 µm target particle size was reached with approximately three-fold less mechanical energy input compared to ambient temperature. Interestingly, the composition of particles differed depending on grinding temperatures: the multi-layered composite structure of bran ended up in particles from cryogenic conditions, denoting a random distribution of crossing cracks during the process, whereas the mechanical load in ambient conditions caused a concomitant breakdown and dissociation of the tissue layers, leading to a variety of distinct particle compositions as illustrated in the depiction of Figure 4. It was hypothesized that, at low temperature, all tissues exhibit identical mechanical properties, then bran behaves like a single vitreous material, while at ambient temperature, tissues differ in their elasto-plastic properties which results in the detachment of the layers under the mechanical stress. It was observed that lipids in the so-called testa bran layer exhibited a glass-transition temperature around −50 °C, which is consistent with a differential susceptibility to grinding [64]. Silva (2013) observed that processing wheat straw at −100 °C in a sieve-based impact grinder reduced the size of particles by ~20% further than at ambient temperature, whereas a ~45% improvement was obtained in a ball-mill [65]. This was attributed to a fast ejection from the milling chamber through the sieve in the first case because of the elongated shape of straw particles. Cryogenic conditions also had the advantage to correct the negative milling effect of high moisture content (~20%) by freezing absorbed water and then removing its plasticizing effect. However, due to the limited gains obtained, cryo-milling was not considered worthwhile for straw fine milling, considering the investments and functioning costs. In the industry, cryo-milling is reserved for processing matters ending to rather high added-value products, for example, adhesives and waxes to avoid deformability and stickiness, explosive materials to keep temperatures below the ignition range, spices to improve the retention of etheric oils and the prevention of oxidation and rancidity, together with higher throughputs and mechanical power savings.

## 5. Discussion: Choice of Milling Equipment

For a given biomass, the choice of a milling equipment over another and the process parameters will influence the properties of the produced powders and the energy consumption. This choice is often crucial but remains difficult as numerous factors need to be considered. For example, it is important to take into consideration: (non-exhaustive list):

- The intended application and the specifications for the ground powder
- The total energy consumption of the device and the power requirement in relation to the production time
- The frequent changes in raw materials to be milled and their properties
- The operating conditions (batch or continuous, inventory of raw and ground materials)
- The investment and operational costs
- The overall dimensions of the installation
- The cleaning procedure and the possible contamination of the biomass materials by the grinder or milling media
- The technical feasibility and the economic possibility of additional cost of a thermic pretreatment

However, it is generally impossible to meet all specifications at the same time and the factors need to be prioritized according to the main constraints and the intended applications. In the case of fine and/or ultra-fine milling, it is often more cost-effective to realize successive milling with different technologies [65]. In addition, it is sometime very difficult to reach the targeted size with an acceptable energy consumption only with milling technologies. In this case, a sorting step (sieving, air classification, or electrostatic separation) can be added to the process diagram. The technologies that can be used and some examples of utilization for lignocellulosic biomass have been reported by Mayer-Laigle et al. in 2017 [66].

**Figure 5:**
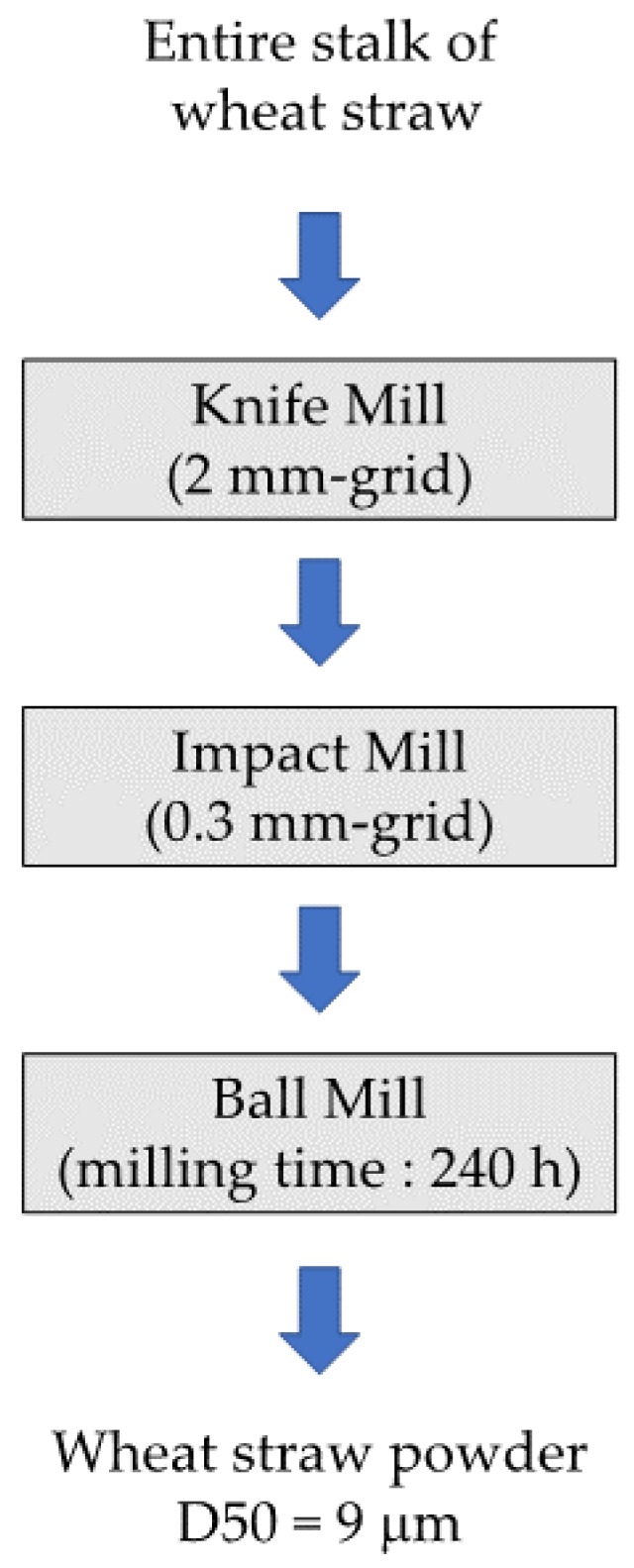
Process diagram for the milling of wheat straw up to a median particle size below 10 µm suitable for bioethanol production and charge for bio-composite production.

In order to help the reader in the process of selecting a grinder, we present here an example of a diagram process (Figure 5) for the ultrafine milling of wheat straw for bioethanol production and bio-composite for packaging application by explaining at each step the technological choices made in regard to the specifications [16,67].

For each of these applications, it has been demonstrated that a high particle size reduction until a median size (d50) below 10 µm was suitable to increase the conversion yield and the fiber–matrix interaction, respectively. Wheat straw is a fibrous raw material which presents an anisotropy in the direction of the stalk [68]. The comminution in this direction requires shear and cuts as mechanical stresses in order to breakdown the stalk and reduce the anisotropy of the materials. Thus, the choice of a knife-mill equipped with a grid of 2 mm seems to be more appropriate in comparison to a jaw crusher or hammer mill which enhance impact as the main mechanical stress, leading to higher energy consumption [21]. At the end of the first milling step, the median particle size d50 is equal to 760 µm. This size is bigger than those that can be obtained by other one-step processes, such as steam explosion, but the particle size distribution obtained is tightened, highlighting a greater uniformity in the ground particles. To continue the particle size reduction, greater forces need to be applied and impact-mill with a sieving grid of 0.3 mm has been selected. As the anisotropy of the wheat straw has been reduced by the first milling step, this technology proved its efficiency and allowed grinding of particles to d50 of approximatively 100 µm. The centrifugal-mill, in which shear is the dominant force, leads to slightly smaller particles but with a low feed rate. The choices therefore prioritized the feasibility and the total energy consumption [16]. In the final milling step, the aim is to divide by ten the d50 of the particles. In this case, the sieve-based grinding was not possible. Indeed, the powder tends to clog the holes of the sieve below 100 µm. Two technologies may be envisioned: jet-milling or ball-milling. The studies show that to reach a d50 of 10 µm, the milling time in batch ball-mill is very long (up to 240 h) in comparison to jet-mill (high power continuous mill). As a result, the associated energy consumption could be greater for a ball-mill than for a jet-mill. However, ball-milling strongly reduces the crystallinity index of cellulose in the ground material and conducts to greater sugar release during the enzymatic degradation for bioethanol production. On the other hand, this ground powder exhibits a greater hydrophobicity which favors the interfacial adhesion between vegetal fiber and the matrix in bio-composite applications. In both cases, the specifications for the ground powder have motivated the final choice of the grinding technologies.

At the industrial scale, the choice of a milling technology must also take into consideration security and health issues, particularly in the case of ultra-fine powders, and integrate the specific regulation of each country in this field. While not exhaustive, the aim of the following paragraph is to give some informative elements on this subject.

## 6. Recommendations Concerning the Health and Security Issues Related to the Milling and Handling of Ultrafine Biomass Powders

Even though for most applications a sufficient target particle size for lignocellulosic fine grinding is generally around 100 µm, inevitably, a range of finer dust is generated during processing and can lead to health and security risks. Although they are limited at the laboratory-scale because the amount of powder handled is small, they cannot be neglected at the industrial scale and must be considered as soon as possible in the scale-up procedures as they can impact technological choices and induce the implementation of specific actions.

### 6.1. Health Risk

The health risks posed by biomass in the form of dust come from both the physico-chemical nature of the particles and their size. As particles become smaller, the risk becomes greater.

According to “Glossary of Atmospheric Chemistry Terms” [69], dust concerns solid particles with aerodynamic diameters below 100 µm or with a deposition rate lower than 0.25 m·s^−1^ [70]. Three classes of atmospheric dust are considered from a sanitary point of view: between 100 and 10 µm, so-called “total dust” retained in the nasal cavity, between 10 and 5 µm, particles entering the trachea, bronchi, and bronchioles, and between 5 and 0.5 µm, very fine dust which deposits in pulmonary alveoli (below 0.5 µm, particles are considered as behaving as a gas) [71].

Wood dust can provoke respiratory and cutaneous pathologies. Repeated deposits of dust in the upper respiratory tract can be at the origin of primo-cancers of nasal cavities and associated sinus. Wood dusts are classified as a group I carcinogen (recognized as carcinogen for humans) by the International Agency for Research on Cancer. Fine dusts reaching the deep lung can result in definitive damages such as pulmonary fibrosis. Wood dust can also be the source of injuries caused by skin and mucosa irritation, and lead to allergic phenomena in some cases (eczema, rhinitis, asthma) [72,73].

A widespread and well-known allergy to fine dust is the so-called baker’s asthma which affects people exposed to flour production, a fine milling process of grains and seeds. Baker’s asthma is the first profession-linked disease in Europe (20% of total professional diseases) [74]. The disease appears after exposure to inhalation of fine flour particles from cereals (wheat, rye, etc.) and leguminosae (soya, lupine, etc.) suspended in the atmosphere. The severity of the disease seems more linked to the intensity of exposure than to its duration. The main allergens evidenced in this pathology belong to the albumin-globulin family that is the major water-soluble protein fraction of flours. The allergens are generally enzymes such as acetyl-CoA oxidase, several peroxidases, alpha-amylase inhibitors, but all involved allergens are far from being identified.

The general limit values of professional exposure to dusty atmospheres over an 8 h period are 10 mg·m^−3^ for total dust and 5 mg·m^−3^ for alveoli dust. The specific health effects of certain dusts (allergenicity, carcinogenicity, recognized source of professional disease) justify even lower maximum exposure values (e.g., 1 mg·m^−3^ for wood dust in France, [70]). Paradoxically, in Europe, the professional exposure limit value for hard wood dust is still at 5 mg·m^−3^ but an upcoming directive is about to set up a 3 mg·m^−3^ threshold. During processing, collective protection by dust uptake as close to the source as possible and atmosphere purification by filtration must systematically be implemented together with individual protection of operators in the form of breathing masks with appropriate filters.

### 6.2. Atex Explosion Hazard

In addition to health risks, fine biomass particles in sufficient quantity and concentration can be a source of rapid combustion, taking the form of a deflagration if it occurs in a confined space [75]. The explosion is initiated by the rapid combustion of a cloud of flammable particles, the oxygen required for combustion being mostly supplied by atmospheric air. If the combustion is so rapid that the pressure increases faster than it can be dissipated, a destructive explosion can occur even in an unconfined cloud. If all particles below 500 µm can potentially provoke an explosion, the risk is higher with the finest particles because they stay in suspension for a longer time. The violence and the speed of the explosion are related to the particle size, the energy release due to combustion regarding the degree of confinement, and heat losses [76]. The combustion occurs if the concentration of powder in the cloud is within the explosive range defined by the Lower and Upper Explosive Limit (LEL and UEP, respectively) and if there is an ignition source. The ignition source is often a spark discharge due to friction between powder or wall of the equipment. In this case the amount of energy needed to start the reaction has to be superior to the Minimum Ignition Energy (MIE) of the biomass considered. A self-inflammation of the cloud or of the biomass in layer can also occur if the temperature is superior to the Minimum Ignition Temperature (MIT) of the cloud or biomass layer. It must be noted that in the case of biomass storage, the temperature could increase notably in the bulk powder due to biological degradation [77]. These different values depend on the biomass type in relation to its composition, the particle size, and its water content. While the values for wood flours are fairly well known, there is a lack of data in the literature for other biomasses that do not belong to a structured sector. In this case, their determination requires tests that must be realized by accredited companies. As an example, the LEL for wood flour is around 100 g·m^−3^ [78], and the EMI between 30 and 60 mJ depending on the particle size and the wood species. For wheat straw, Jacobson measured an EMI of 50 mJ [79]. To minimize the explosion hazard, it is possible to add an inerting substance in order to increase the value of the MIE [80]. However, this modifies also the properties of the biomass powder, especially when it is intended for combustion or other energetic applications.

The industrial installations must be zoned according to the frequency and time that an explosive atmosphere may be potentially present in the form of a cloud of combustible dust in air. Three zones must be defined: (i) Zone 20 where the explosive atmosphere is present continuously, for long periods or high frequencies; (ii) Zone 21 where the explosive atmosphere is likely to occur occasionally in normal operation (as for example during a cleaning phase); (iii) Zone 22: where the explosive atmosphere is not likely to occur in normal operation, but if it does occur by accident, it will persist for a short time [81]. Any facilities installed in an ATEX zone must be designed, certified, and maintained according to the regulations. However, the definition of the different zones is qualitative and may be dependent on the person carrying out the study.

## 7. Conclusions

The ultrafine milling of lignocellulosic biomass by dry processing is a key issue in many emerging applications. If numerous efforts have been realized at the laboratory-scale to establish a relation between the properties of the raw materials, their milling behavior, and the properties of the end-product, general laws that can be transposed at the industrial scale are still difficult to identify, particularly concerning the energy consumption. As a result, the choice of a milling process, generally involving several grinding steps, proves to be often difficult, complicated by the fact that milling technologies dedicated for the ultrafine milling of biomass are very few and manufacturers have generally scarce expertise in this field. Although it has been shown that thermic pretreatments can reduce the total energy consumption while improving certain properties of the ground powders, some security and health issues could increase the investment and functioning costs of an industrial plant. In addition, the added value of the end-product often remains low in comparison to other business segments. Thus, an entrepreneur who decides to innovate in this field takes a significant risk and support policies are required to boost the lignocellulosic biomass applications that involve a fine or ultrafine milling step.

Furthermore, research actions centered on the understanding of the lignocellulosic milling behavior via, for example, the implementation of specific sensors to follow the comminution of raw materials during the process will probably help to define a better adequacy of milling equipment for lignocellulosic materials. This knowledge could also enable the development of eco-designed grinders in view of the sustainable development of processes. In summary, basic academic research is more than ever necessary to improve our knowledge of the physical interactions between matter and machine, that can help, together with advanced engineering science, to implement innovative technologies for efficient use of lignocellulosic biomass in the future.

## Figures and Tables

**Table 1:** Operating conditions of the different milling equipment shown in Figure 1.

Figure	Type of Grinder	Mechanical Stresses	Operating Principle	Operating Mode	Targeted Particle Size
1a	Impact mill	Impact and shear	Particles are milled by a tool which rotates between 10,000 and 20,000 rpm. A sieving grid allows the selection of the output particles according to their size.	Continuous	100–1000 µm
1b	Pin mill	Shear	Particles are milled between two pin discs rotating with a high differential speed	Continuous	50–1000 µm
1c	Vibratory ball mill	Compression and attrition	Particles are milled by a milling media put in motion by the vibration of the tank	Batch	10–500 µm
1d	Opposite jet mill	Impact and attrition	Particles are projected in a central point by air-compressed nozzles	Continuous	5–100 µm
